# Correction: Preparation of a colloidal photonic crystal containing CuO nanoparticles with tunable structural colors

**DOI:** 10.1039/c9ra90020a

**Published:** 2019-03-21

**Authors:** Chun-Feng Lai, Yu-Chi Wang, Chia-Jung Wu, Jia-Yu Zeng, Chia-Feng Lin

**Affiliations:** Department of Photonics, Feng Chia University No. 100, Wenhwa Road, Seatwen Taichung 40724 Taiwan chunflai@fcu.edu.tw; Department of Materials Science and Engineering, National Chung Hsing University Taichung 402 Taiwan

Correction for ‘Preparation of a colloidal photonic crystal containing CuO nanoparticles with tunable structural colors’ by Chun-Feng Lai *et al.*, *RSC Adv.*, 2015, **5**, 105200–105205.

Chia-Jung Wu’s name was incorrectly spelled in the published article; the correct version is shown above.

The Royal Society of Chemistry apologises for these errors and any consequent inconvenience to authors and readers.

## Supplementary Material

